# Supplementary material for: Constitutive smooth muscle tumour necrosis factor regulates microvascular myogenic responsiveness and systemic blood pressure
Source: Nat Commun. 2017 Apr 5;8:14805. doi: 10.1038/ncomms14805 (PMC5382284; doi:10.1038/ncomms14805)
Supplement: Supplementary Information — Supplementary Figures and Supplementary Tables. [file ncomms14805-s1.pdf]

SUPPLEMENTARY INFORMATION FILE

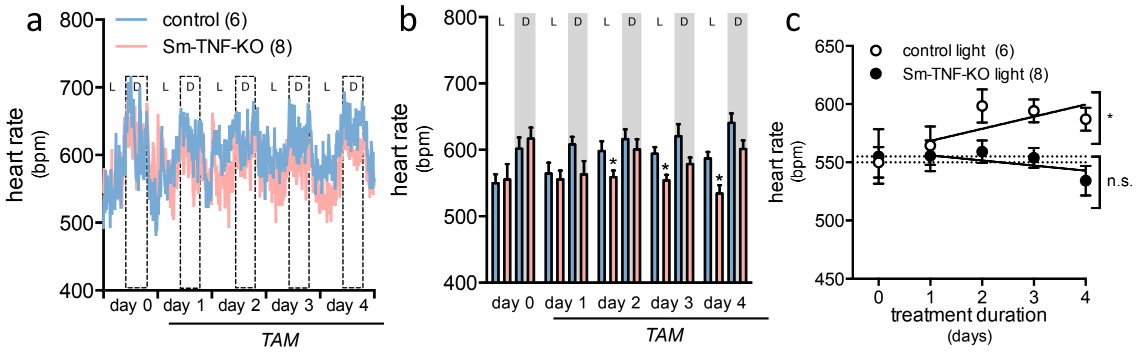

SUPPLEMENTARY FIGURE 1: Heart rate in tamoxifen-inducible smooth muscle-specific *Tnf* knockout mice. Mice expressing inducible Cre recombinase within their smooth muscle (SMMHC-*Cre*ER^{T2}) and carrying either the wild type *Tnf* gene (*Tnf*$^{wt/wt}$, control) or a floxed *Tnf* gene (*Tnf*$^{fl/fl}$) were treated with tamoxifen (TAM, 1 mg/day), resulting in a smooth muscle cell specific *Tnf* gene knockout (Sm-TNF-KO). Day 0 represents the untreated control condition. (**a**) Continuous radiotelemetric heart rate measurements in non-anaesthetized mice; displayed are average raw tracings; light and dark phases are indicated as “L” and “D”, respectively. (**b**) Phase analysis. Blue bars indicate control, pink bars indicate Sm-TNF-KO, and grey shading indicates dark phase. (**c**) Light phase heart rate measurements during tamoxifen treatment. Numbers in parentheses indicate numbers of animals per group. All data are mean ± s.e.m.; * $P<0.05$ in Student’s unpaired *t*-test (**b**); * $P<0.05$ and n.s., non-significant in repeated-measures one-way ANOVA (**c**).

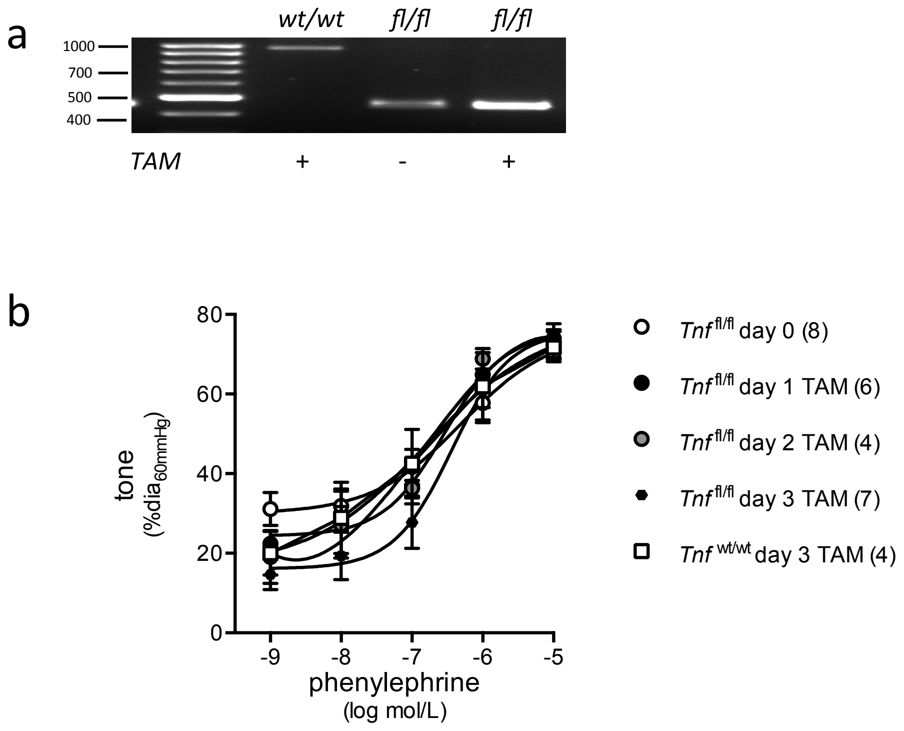

SUPPLEMENTARY FIGURE 2. Genotyping and myogenic responses of tamoxifen-inducible smooth muscle-specific *Tnf* knockout mice. **(a)** Representative image of PCR cleavage product in aortae isolated from mice expressing inducible Cre recombinase within their smooth muscle (SMMHC-*Cre*ER^{T2}) and carrying either the wild type *Tnf* gene (*Tnf*$^{wt/wt}$, control) or a floxed *Tnf* gene (*Tnf*$^{fl/fl}$) untreated (-) or treated with tamoxifen (TAM, 1 mg/day for 3 days, +). **(b)** Pressure myography measurements of isolated mouse cremaster skeletal muscle resistance arteries from control and smooth muscle cell specific *Tnf* gene knockout mice (Sm-TNF-KO). Shown are phenylephrine-stimulated responses. Numbers in parentheses indicate numbers of arteries per group. Data are mean ± s.e.m. * $P<0.05$ for one-way ANOVA and Dunnett's *post-hoc* test compared to Sm-TNF-KO at 0 days.

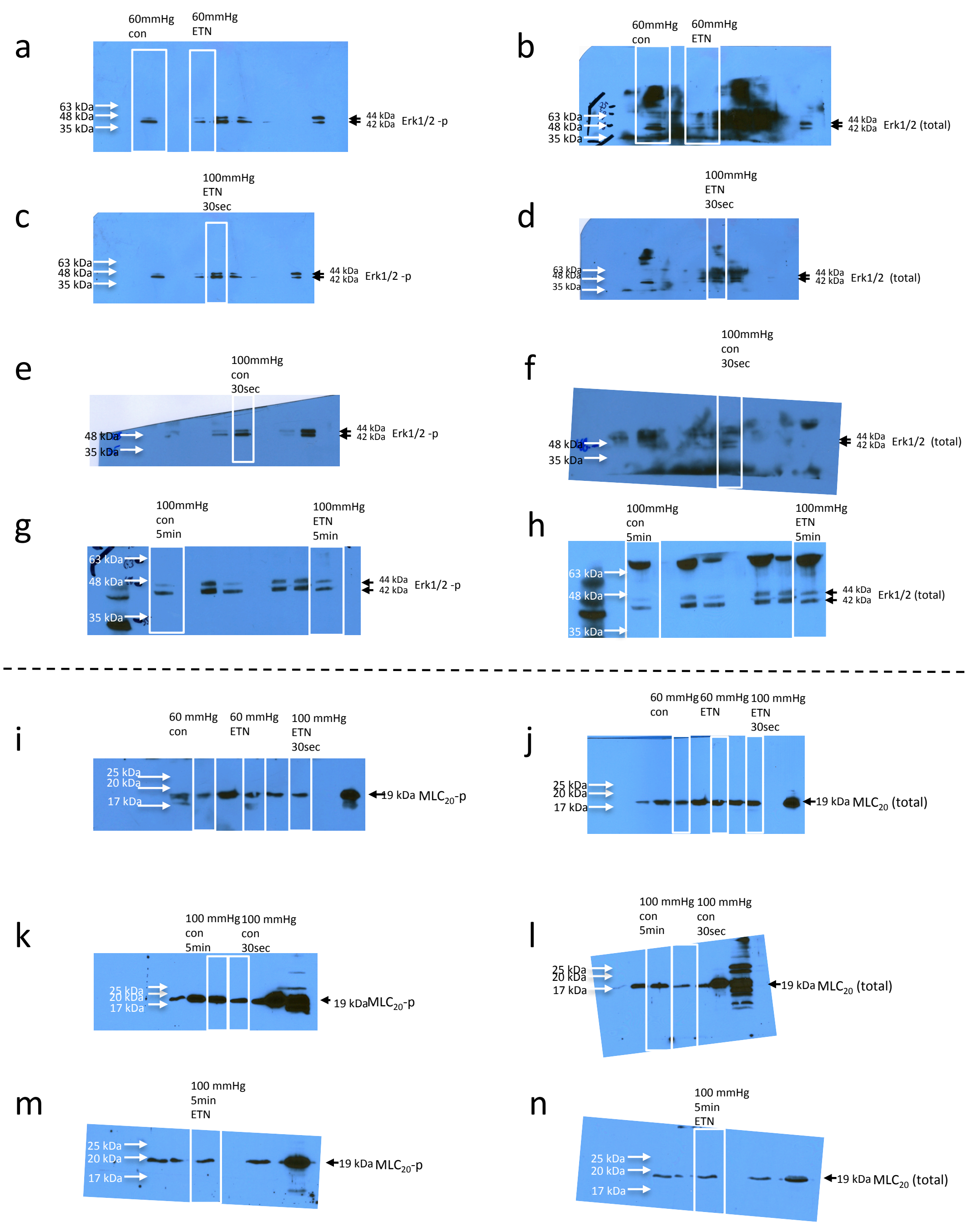
a
60mmHg con
60mmHg ETN
63 kDa
48 kDa
35 kDa
44 kDa
42 kDa
Erk1/2 -p
b
60mmHg con
60mmHg ETN
63 kDa
48 kDa
35 kDa
44 kDa
42 kDa
Erk1/2 (total)
c
100mmHg ETN 30sec
63 kDa
48 kDa
35 kDa
44 kDa
42 kDa
Erk1/2 -p
d
100mmHg ETN 30sec
63 kDa
48 kDa
35 kDa
44 kDa
42 kDa
Erk1/2 (total)
e
100mmHg con 30sec
48 kDa
35 kDa
44 kDa
42 kDa
Erk1/2 -p
f
100mmHg con 30sec
48 kDa
35 kDa
44 kDa
42 kDa
Erk1/2 (total)
g
100mmHg con 5min
100mmHg ETN 5min
63 kDa
48 kDa
35 kDa
44 kDa
42 kDa
Erk1/2 -p
h
100mmHg con 5min
100mmHg ETN 5min
63 kDa
48 kDa
35 kDa
44 kDa
42 kDa
Erk1/2 (total)
i
60 mmHg con
60 mmHg ETN
100 mmHg ETN 30sec
25 kDa
20 kDa
17 kDa
19 kDa MLC20-p
j
60 mmHg con
60 mmHg ETN
100 mmHg ETN 30sec
25 kDa
20 kDa
17 kDa
19 kDa MLC20 (total)
k
100 mmHg con 5min
100 mmHg con 30sec
25 kDa
20 kDa
17 kDa
19 kDa MLC20-p
l
100 mmHg con 5min
100 mmHg con 30sec
25 kDa
20 kDa
17 kDa
19 kDa MLC20 (total)
m
100 mmHg 5min ETN
25 kDa
20 kDa
17 kDa
19 kDa MLC20-p
n
100 mmHg 5min ETN
25 kDa
20 kDa
17 kDa
19 kDa MLC20 (total)

SUPPLEMENTARY FIGURE 3. Uncropped western blot images of ERK1/2 and $\mathbf{MLC_{20}}$ in mouse cremaster arteries. (**a**) phospho-ERK1/2 and (**b**) total ERK1/2 in mouse cremaster arteries exposed to 60 mmHg pressure under control conditions (con) and in the presence of etanercept (ETN). (**c**) phospho-ERK1/2 and (**d**) total ERK1/2 in mouse cremaster arteries exposed to 100 mmHg pressure for 30 seconds in the presence of ETN. (**e**) phospho-ERK1/2 and (**f**) total ERK1/2 in mouse cremaster arteries exposed to 100 mmHg pressure for 30 seconds under control conditions. (**g**) phospho-ERK1/2 and (**h**) total ERK1/2 in mouse cremaster arteries exposed to 100 mmHg pressure for 5 min under control conditions and in the presence of ETN.
(**i**) phospho-MLC_{20} and (**j**) total MLC_{20} in mouse cremaster arteries exposed to 60 mmHg pressure under control conditions and in the presence of ETN at 60 and 100 mmHg for 30 seconds. (**k**) phospho-MLC_{20} and (**l**) total MLC_{20} in mouse cremaster arteries exposed to 100 mmHg pressure under control conditions for 5 min and 30 s, respectively. (**m**) phospho-MLC_{20} and (**n**) total MLC_{20} in mouse cremaster arteries exposed to 100 mmHg pressure in the presence of ETN. Rectangular boxes indicate area cropped for representative image.

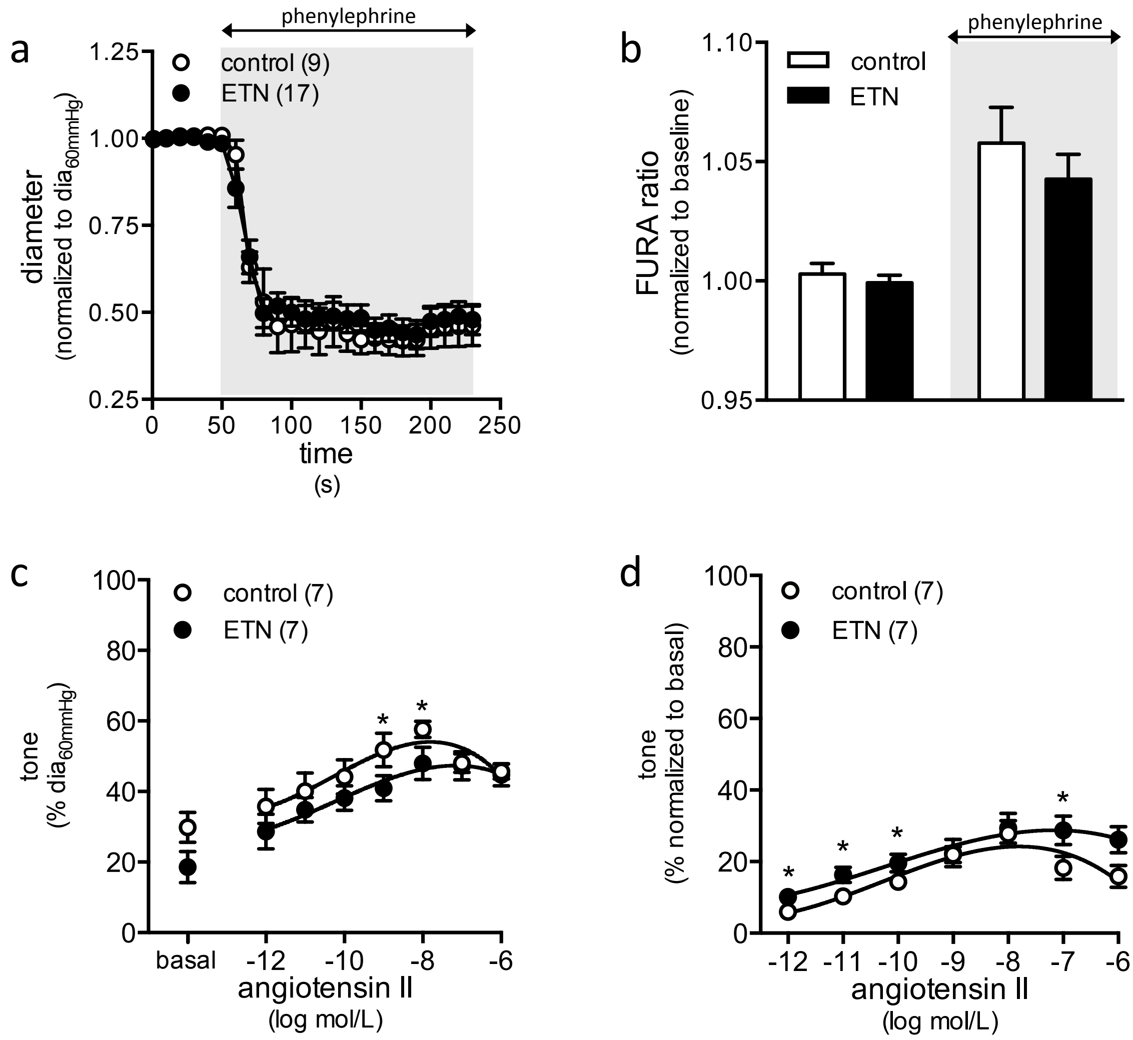

SUPPLEMENTARY FIGURE 4. Phenylephrine- and angiotensin II-stimulated responses of mouse skeletal muscle cremaster muscle resistance arteries with etanercept. Phenylephrine-stimulated (10 μmol/L, grey shading) vasoconstriction in isolated arteries in the presence of etanercept (ETN; 300 μg/mL) or normal buffer (control). **(a)** Diameter responses are normalized to baseline diameter (10 s average; control: 55±5, ETN: 63±3 μm). **(b)** Intracellular Ca^{2+} levels (control: n =5 arteries in 0 and 5 in 10 μmol/L PE; ETN: n = 7 arteries in 0 and 7 in 10 μmol/L PE). **(c)** Angiotensin II-stimulated vasoconstriction in isolated arteries in the presence of ETN (300 μg/mL) or normal buffer (control). Basal indicates tone prior to addition of angiotensin II. **(d)** Angiotensin II-stimulated vasoconstriction normalized to basal tone (control: 30±4, ETN: 19±4 % tone). Numbers in parentheses indicate number of arteries per group. Data are mean ± s.e.m. * $P<0.05$ for unpaired Student's *t*-test (**a,b**) and paired Student's *t*-test (**c,d**).

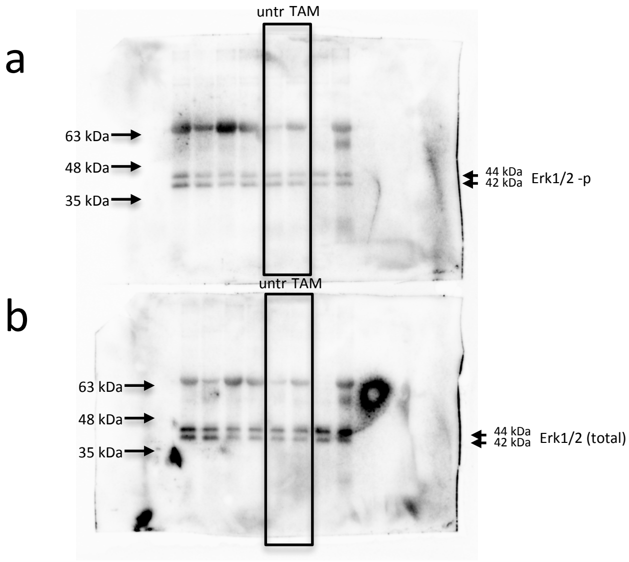

SUPPLEMENTARY FIGURE 5. Uncropped images of ERK phosphorylation in cremaster arteries from smooth muscle *Tnf* knockout mice. Cremaster muscle arteries were excised from tamoxifen treatment (TAM, 1mg/day for 3 days) in Sm-TNF-KO mice and untreated controls. (**a**) Uncropped western blot image for phospho-ERK1/2 and (**b**) total ERK1/2.

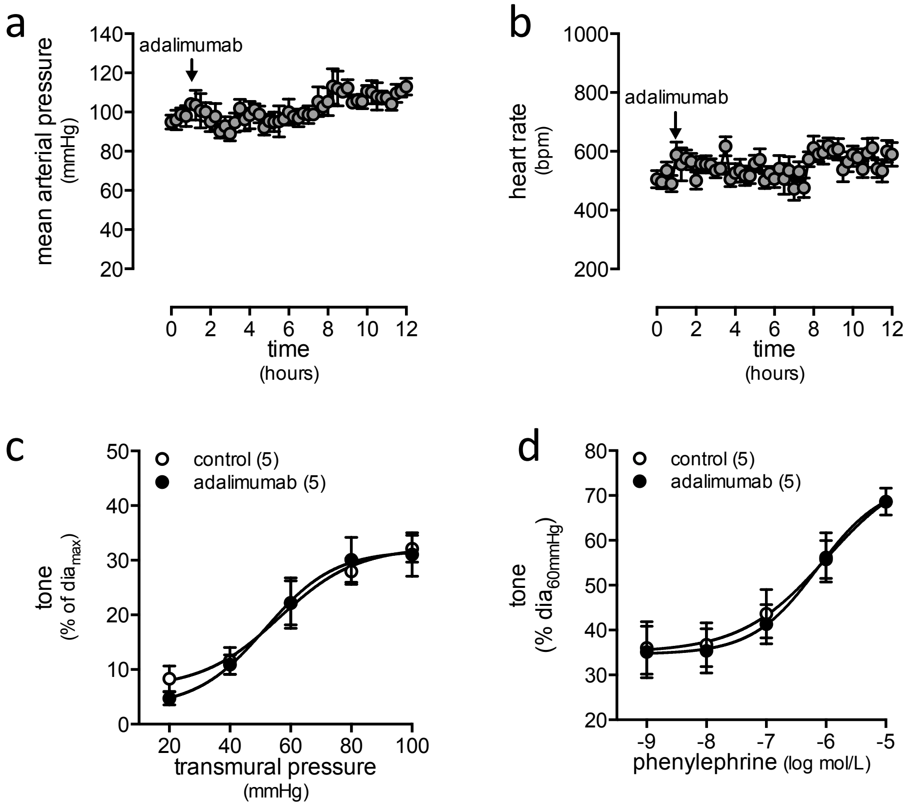

SUPPLEMENTARY FIGURE 6. Hemodynamic and microvascular responses to adalimumab. Radiotelemetric hemodynamic measurements of naive wild type mice injected with (adalimumab; 20 mg/kg i.p., arrow, n=8 mice). Mean arterial pressure (**a**) and heart rate (**b**) in response to adalimumab. Microvascular responses were conducted in isolated cremaster skeletal muscle resistance arteries. (**c**) Myogenic responsiveness in the absence and presence of adalimumab (300 µg/mL *in vitro*). (**d**) Phenylephrine-stimulated vasoconstriction in the absence and presence of adalimumab (300 µg/mL *in vitro*). Numbers in parentheses indicate number of arteries per group. All data are mean ± s.e.m. All data are non-significant in a one-way repeated-measures ANOVA and Dunnett's *post-hoc* test relative to 0 hours (**a,b**) and an unpaired Student's *t*-test (**c,d**).

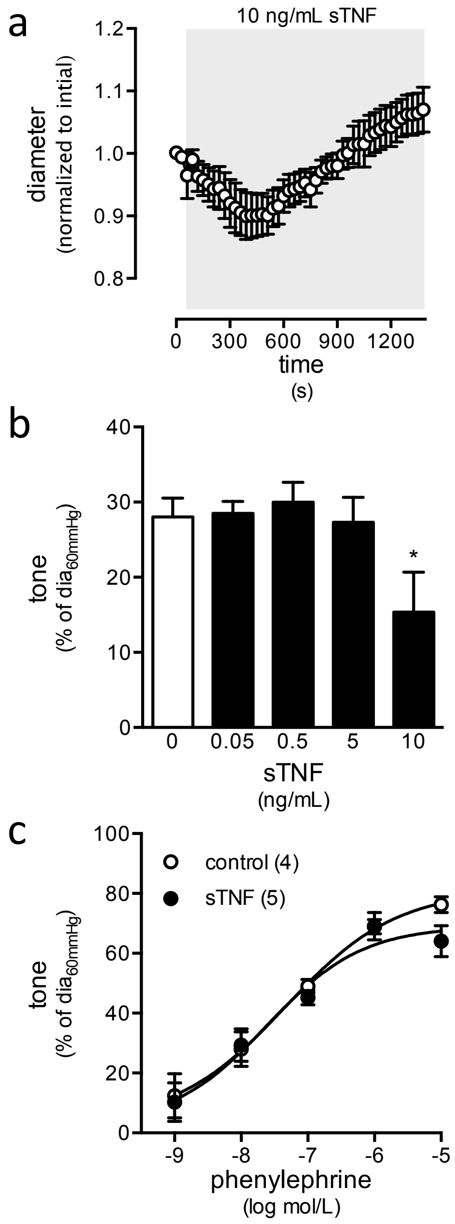

SUPPLEMENTARY FIGURE 7. Microvascular responses in mouse cremaster skeletal muscle resistance arteries to exogenously applied soluble TNF. (a) Shown are normalized diameter changes in cremaster skeletal muscle resistance arteries following incubation with human recombinant soluble TNF (sTNF, 10 ng/mL *in vitro*, shaded area, n=5 arteries). Data are normalized to baseline diameter (10 s average: 61±6 µm). **(b)** Myogenic tone at 60 mmHg following incubation with sTNF (n= 7 arteries at 0, 5 at 0.05, 6 at 0.5, 6 at 5, and 6 at 10 ng/mL sTNF). **(c)** Phenylephrine-stimulated vasoconstriction in the absence and presence of sTNF (10 ng/mL, *in vitro*). Numbers in parentheses indicate number of arteries per group. Data are mean ± s.e.m. * $P < 0.05$ for one-way ANOVA and Dunnett's *post-hoc* test compared to 0 ng/mL **(b)**. An unpaired Student's *t*-test at each concentration was non-significant (**c**).

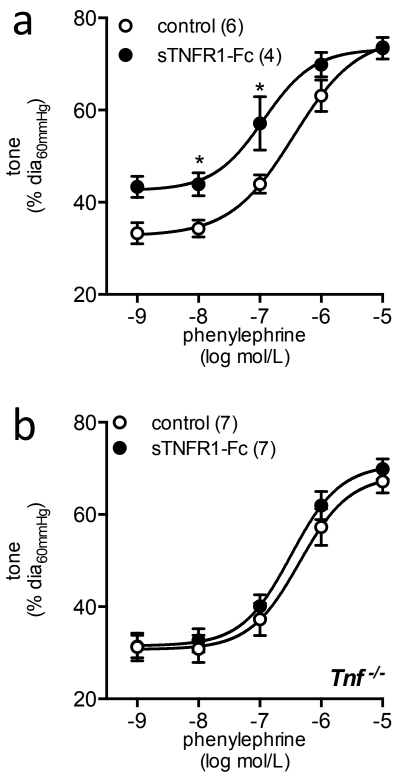

SUPPLEMENTARY FIGURE 8. Microvascular responses in mouse cremaster skeletal muscle resistance arteries to sTNFR1-Fc. Shown are normalized diameter changes in cremaster skeletal muscle resistance arteries following isolated from **(a)** wild type and **(b)** *Tnf*$^{-/-}$ at 60 mmHg transmural pressure in the absence or presence of soluble TNFR1 fragment (sTNFR1-Fc, 100 ng/mL, arrow). Data are normalized to baseline diameter (10 s average; in wild type, control: 56±5, sTNFR1-Fc: 54±4; in *Tnf*$^{-/-}$, control: 51±4, sTNFR1-Fc: 56±2 μm). The upward shift in phenylephrine-stimulated tone is likely due to the elevated myogenic responsiveness induced just prior to the application of phenylephrine. Numbers in parentheses indicate number of arteries per group. Data are mean ± s.e.m. * P < 0.05 for unpaired Student's *t*-test (**a, b**).

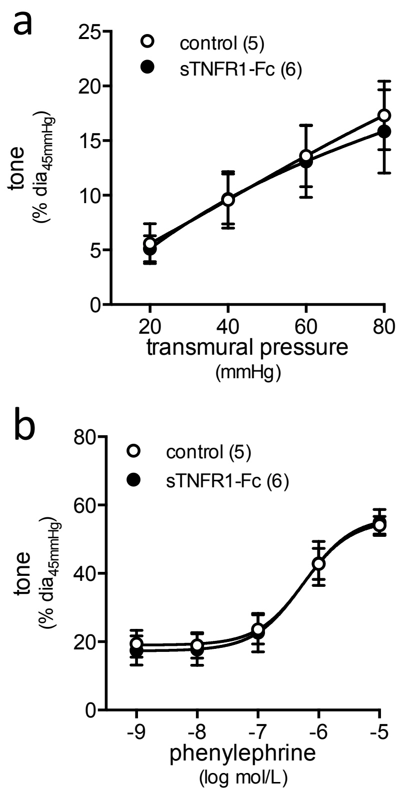

SUPPLEMENTARY FIGURE 9. Microvascular responses in mouse olfactory cerebral arteries to sTNFR1-Fc. Pressure myography of olfactory cerebral resistance arteries isolated from wild type mice. **(a)** Myogenic responses in the absence and presence of soluble TNFR1 fragment (sTNFR1-Fc, 100 ng/mL *in vitro*). **(b)** Phenylephrine-stimulated vasoconstriction in the absence and presence of sTNFR1-Fc. Numbers in parentheses indicate number of arteries per group. All data are mean ± s.e.m. An unpaired Student's *t*-test at each pressure (**a**) and dose (**b**) was non-significant.

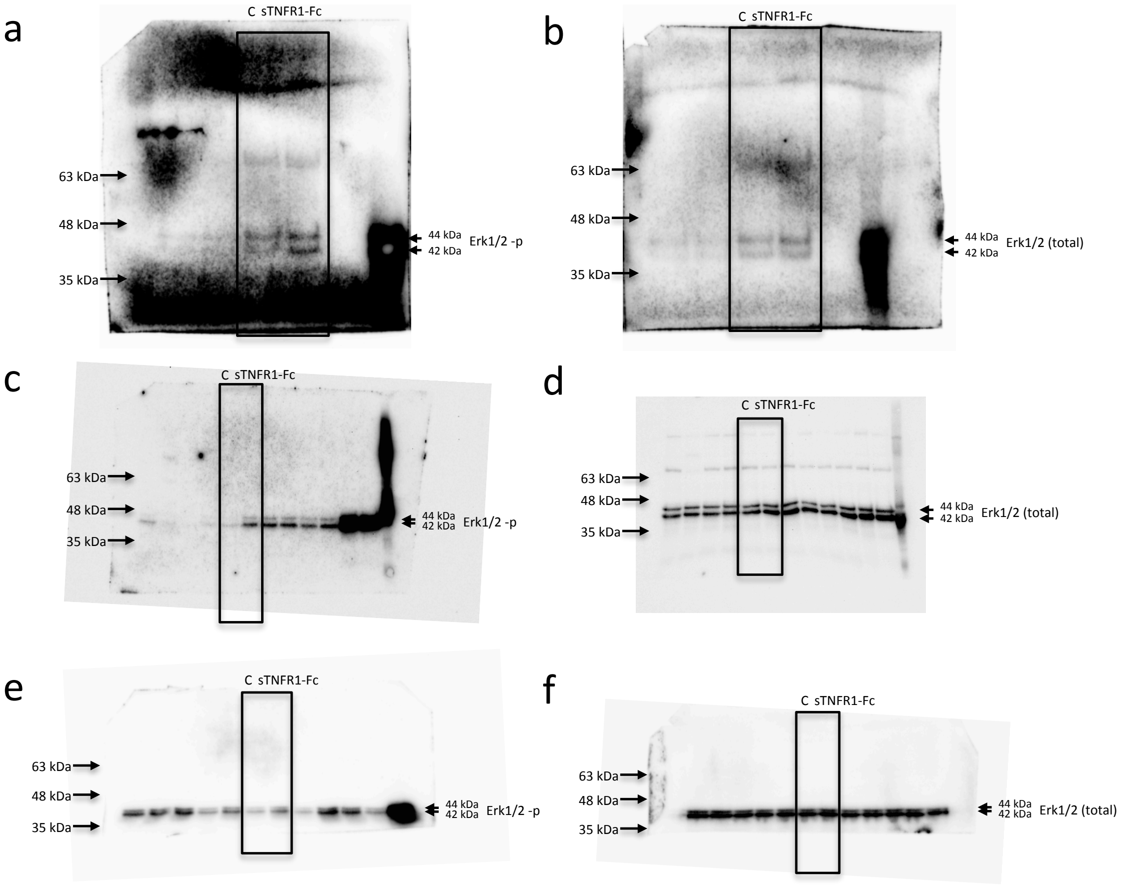

SUPPLEMENTARY FIGURE 10. Uncropped western blot images of ERK1/2 in mouse cremaster arteries and smooth muscle cell cultures. (**a**) phospho-ERK1/2 and (**b**) total ERK1/2 in mouse cremaster arteries under control conditions (C) and in the presence of the soluble TNFR1 fragment (sTNFR1-Fc). (**c**) phospho-ERK1/2 and (**d**) total ERK1/2 in cultured mouse vascular smooth muscle cells under control conditions and in the presence of sTNFR1-Fc. (**e**) phospho-ERK1/2 and (**f**) total ERK1/2 in cultured human coronary artery vascular smooth muscle cells under control conditions and in the presence of sTNFR1-Fc. Rectangular boxes indicate area cropped for representative image.

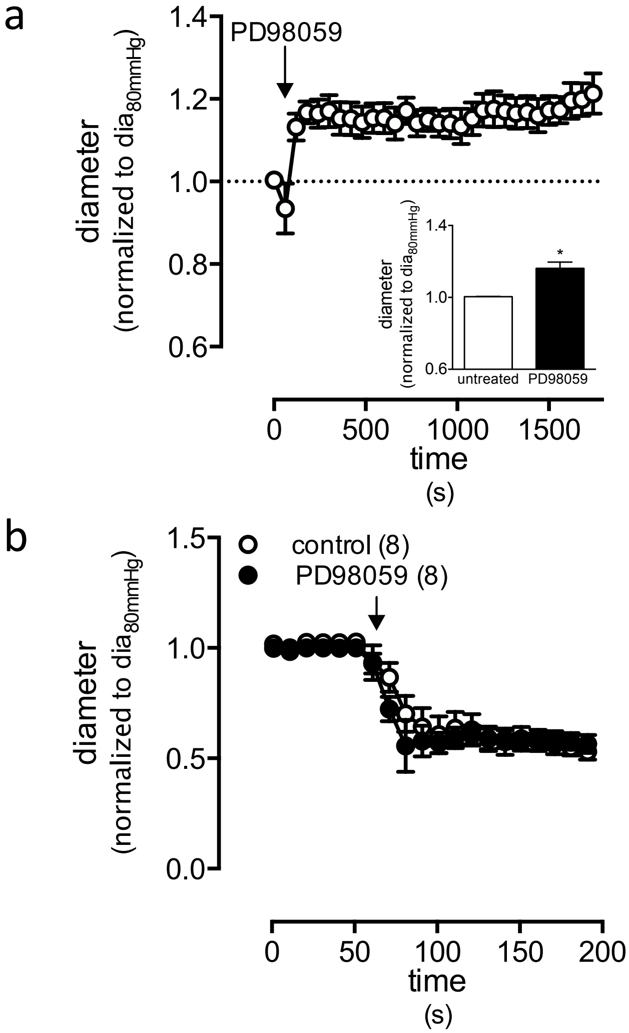

SUPPLEMENTARY FIGURE 11. Treatment of mouse cremaster skeletal muscle resistance arteries with PD98059. (a) Direct addition of PD98059 (10 µmol/L *in vitro*, arrow, n= 8 arteries) at 80 mmHg transmural pressure. Data are normalized to baseline diameter (10 s average: 45±3 µm). Inset; statistical comparison of 1 s versus 121-1741 s (both control and PD98059: n= 8 arteries per group). **(b)** Phenylephrine-stimulated vasoconstriction (10 µmol/L, arrow) in the absence or presence of PD98059 (10 µmol/L). Data are normalized to baseline diameter (10 s average control: 63±9, PD98059: 52±4 µm). Numbers in parentheses indicate number of arteries per group. Data are mean ± s.e.m. * $P < 0.05$ for paired Student's *t*-test (**a** inset, **b**).

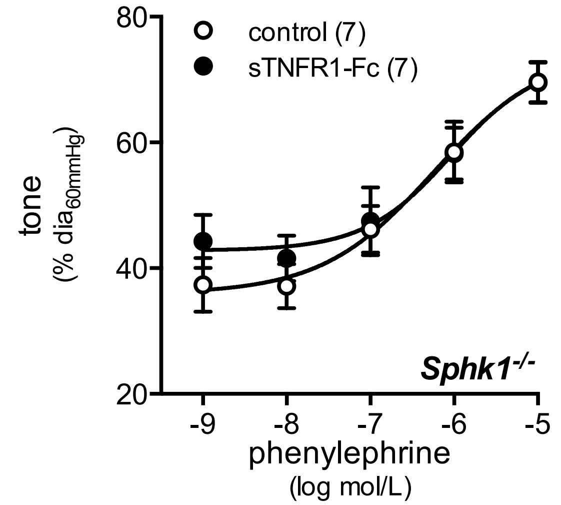

SUPPLEMENTARY FIGURE 12. Microvascular responses in *Sphk1*$^{-/-}$ mouse cremaster skeletal muscle resistance arteries to sTNFR1-Fc. Phenylephrine-stimulated vasoconstriction in the absence or presence of sTNFR1-Fc (10 ng/mL, *in vitro*). Numbers in parentheses indicate number of arteries per group. Data are mean ± s.e.m. An unpaired Student's *t*-test at each dose was non-significant.

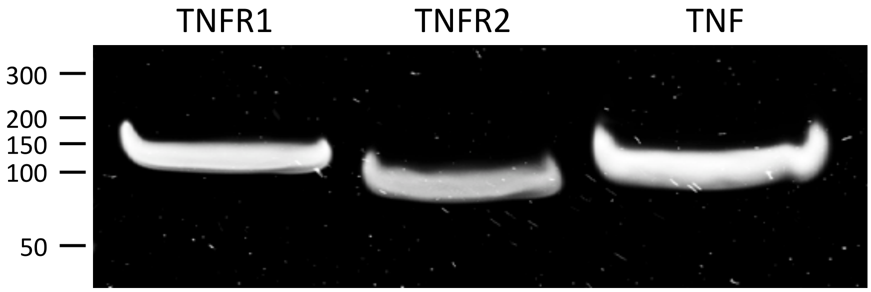

SUPPLEMENTARY FIGURE 13. TNF and TNF receptor expression in wild type mouse cremaster arteries. Representative image of endpoint PCR for TNF receptor 1 (p55, TNFR1), TNF receptor 2 (p70, TNFR2) and TNF isolated mouse cremaster skeletal muscle resistance arteries.

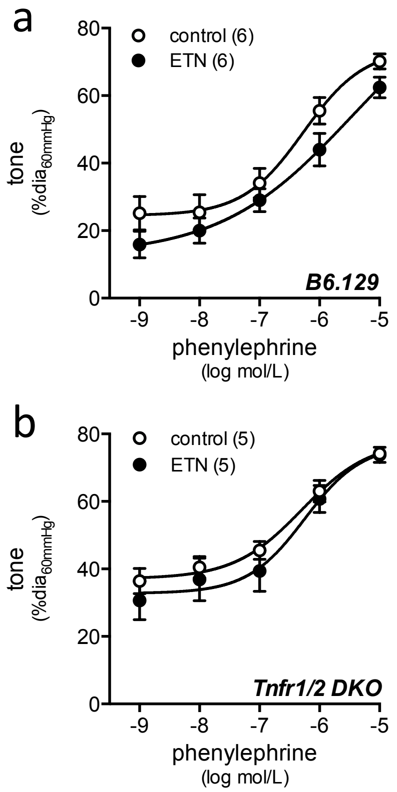

SUPPLEMENTARY FIGURE 14. Phenylephrine-induced constriction in wild type and *Tnfr1/2 DKO* mouse cremaster muscle arteries to etanercept. (**a**) Phenylephrine-stimulated vasoconstriction in arteries isolated from B6.129 wild type control mice in the absence and presence of etanercept (ETN, 300 µg/mL *in vitro*). (**b**) Phenylephrine-stimulated vasoconstriction in arteries isolated from *Tnfr1/2 DKO* mice in the absence and presence of etanercept (ETN, 300 µg/mL *in vitro*). Numbers in parentheses indicate number of arteries per group. All data are mean ± s.e.m. * $P<0.05$ for an unpaired Student's *t*-test.

	MAP (mmHg)	CO (mL/min)	TPR (mmHg/mL/min)	SBP (mmHg)	DBP (mmHg)	HR (bpm)	SV (μL)	EF (%)
control (n=8)	74.2±2.11	**23.0±0.49**	**3.22±0.08**	93.3±2.60	64.9±1.82	**511.6±12.72**	45.3±1.72	65.5±1.40
Sm-TNF-KO (n=6)	75.8±1.49	**27.6±1.42***	**2.78±0.11***	96.0±2.02	65.5±1.28	**557.1±13.83***	49.4±1.80	67.1±0.65

SUPPLEMENTARY TABLE 1. Hemodynamic responses in Sm-TNF-KO mice. Measurements from Sm-TNF-KO and control mice following 3 days of tamoxifen treatment. MAP, mean arterial pressure. CO, cardiac output. TPR, total peripheral resistance. SBP, systolic blood pressure. DBP, diastolic blood pressure. HR, heart rate. SV, stroke volume. EF, ejection fraction. Data are mean ± s.e.m. * $P < 0.05$ for unpaired Student's *t*-test.

	HW (mg)	Sd (mm)	Dd (mm)	ESV (μL)	EDV (μL)	dP/dt max (mmHg/sec)	dP/dt min (mmHg/sec)	BW (g)
control (n=8)	116.6±5.6	2.57±0.09	3.98±0.08	24.2±2.05	69.5±3.47	**8831±356**	**-8502±626**	27.7±0.78
Sm-TNF-KO (n=6)	113.7±6.8	2.58±0.04	4.08±0.06	24.3±1.02	73.7±2.63	**11002±587***	**-10357±423***	28.0±0.68

SUPPLEMENTARY TABLE 2. Cardiac parameters in Sm-TNF-KO mice. Measurements from Sm-TNF-KO and control mice following 3 days of tamoxifen treatment. HW, heart weight. Sd, systolic diameter. Dd, diastolic diameter. ESV, end systolic volume. EDV, end diastolic volume. dP/dt max, maximum developed pressure. dP/dt min, minimum developed pressure. BW, body weight. Data are mean ± s.e.m. * $P < 0.05$ for unpaired Student's *t*-test.

Parameter	Cardiac patients	Lumbar patients
n	4	4
Sex, male/female	4/0	3/1
Age, y	61±2	58±3
LVEF, grade	1 (normal)	N/A
**Conditions**		
Hypertension, n	4 (100%)	2 (50%)
Hyperlipidemia, n	4 (100%)	1 (25%)
Diabetes, n	2 (50%)	0 (0%)
History of smoking, n	2 (50%)	3 (75%)
Abdominal obesity, n	1 (25%)	0 (0%)
Myocardial infarction, n	0 (0%)	1 (25%)
Previous bypass graft, n	0 (0%)	1 (25%)
**Medications**		
ACE inhibitor, n	3 (75%)	1 (25%)
B blocker, n	3 (75%)	2 (50%)
Ca2+ channel blocker, n	2 (50%)	0 (0%)
Nitric oxide donor, n	1 (25%)	0 (0%)
Statin, n	4 (100%)	2 (50%)
Anti-diabetic treatment, n	2 (50%)	0 (0%)
Diuretic, n	1 (25%)	1 (25%)
TNF antagonist, n	0 (0%)	0 (0%)

SUPPLEMENTARY TABLE 3. Clinical characteristics of cardiac and lumbar patients. Expressed as number of subjects with parameter and fraction (in %). LVEF, left ventricular ejection fraction. ACE, angiotensin converting enzyme. Data were not statistically compared.
